# Prevalence of hepatitis coinfection and substance use among antiretroviral therapy clinic clients with hazardous alcohol use in Vietnam

**DOI:** 10.1371/journal.pgph.0003744

**Published:** 2024-12-05

**Authors:** Jane S. Chen, Sara N. Levintow, Ha V. Tran, Adams L. Sibley, Natalie A. Blackburn, Teerada Sripaipan, Heidi E. Hutton, Vivian F. Go, Geetanjali Chander

**Affiliations:** 1 Department of Health Behavior, University of North Carolina at Chapel Hill, Chapel Hill, North Carolina, United States of America; 2 Department of Epidemiology, University of North Carolina at Chapel Hill, Chapel Hill, North Carolina, United States of America; 3 RTI International, Research Triangle Park, North Carolina, United States of America; 4 Department of Psychiatry and Behavioral Sciences, School of Medicine, Johns Hopkins University, Baltimore, Maryland, United States of America; 5 Division of General Internal Medicine, University of Washington, Seattle, Washington, United States of America; Kiruddu National Referral Hospital, UGANDA

## Abstract

The confluence of injection drug use (IDU), alcohol consumption, and viral hepatitis increases morbidity among persons living with HIV (PWH). We present a secondary analysis of a randomized controlled trial of alcohol reduction interventions in Thai Nguyen, Vietnam conducted between 2016–2018. We assessed hepatitis B (HBV) and hepatitis C (HCV) coinfection among PWH reporting hazardous alcohol consumption and examined differences in IDU and alcohol use by coinfection status. Participants were ≥18 years old, living with HIV, and reported hazardous alcohol consumption per the WHO Alcohol Use Disorders Identification Test Consumption (AUDIT-C; score ≥4 for men, score ≥3 for women). At enrollment, participants were tested for hepatitis coinfection with HBV surface antigen tests and rapid serological HCV tests. Demographic information, IDU, and recent alcohol consumption were assessed via behavioral survey and 30-day timeline follow back. Fishers Exact and Kruskal-Wallis tests were used for statistical testing. Hepatitis coinfection was common among the 440 enrolled PWH: HCV: n = 355 (81%); HBV: n = 5 (1%); HBV and HCV: n = 37 (8%). Only 10% (n = 43) of participants had no hepatitis coinfection. Among those who tested positive for HBV, 36% had previously been diagnosed with HBV; among those who tested seropositive for HCV, 18% had previously received an HCV diagnosis. History of IDU was higher among those with hepatitis coinfection (HBV or HCV coinfection: 88%; HBV and HCV coinfections: 97%) than those without hepatitis coinfection (7%; p<0.01). Median days of alcohol consumption in the last 30 days was higher among those with coinfection (HBV or HCV coinfection: 20 (Interquartile Range (IQR): 10–30); HBV and HCV coinfections: 22 (IQR: 13–28) than those without hepatitis coinfection (10; IQR: 6–21; p<0.01). The syndemic conditions of HIV, hepatitis, IDU, and alcohol use are deeply entangled and challenging to parse out. Integrated health services are warranted to reduce the risk of liver-related morbidity.

## Introduction

People living with HIV (PWH) experience a disproportionate burden of infection with hepatitis C virus (HCV) and hepatitis B virus (HBV) [1, 2]. In the Asia-Pacific region, research has established a high prevalence of hepatitis coinfection with HIV [3], the association of injection drug use with coinfection, particularly HCV [4–6], and the co-occurrence of hazardous alcohol use in these populations [5]. HIV, hepatitis, drug, and alcohol use each independently impair liver health; their confluence results in high risks of liver-related morbidity and mortality [6, 7]. Understanding health behaviors related to the syndemic conditions can inform potential areas of integrated intervention.

In Vietnam, the prevalence of HBV and HCV coinfection with HIV has been estimated to be 14% and 40%, respectively [8], and HCV coinfection ranges as high as 95–98% in some populations who use injection drugs [9, 10]. However, these prevalence estimates may be outdated [10] and an in-depth examination of drug and alcohol use in these populations is warranted. Additionally, recent work in Vietnam has found discrepancies in HCV coinfection status when self-reports are compared to the medical records of PWH, suggesting that HCV awareness among patients and the screening practices of providers remain inconsistent [11].

In this analysis, we estimate the prevalence of HBV and HCV coinfection among antiretroviral therapy (ART) clinic clients and examine differences in self-reported drug and alcohol use between clients with and without coinfection.

## Materials and methods

### Participants

This analysis is a sub-analysis of a three-arm randomized controlled trial conducted in the northern province of Thai Nguyen, Vietnam between 2016–2018; study recruitment occurred between March 28, 2016 and May 25, 2017. The trial assessed two alcohol consumption reduction interventions (one brief intervention with 2 individual sessions and 2 booster phone calls and one combined intervention with 6 individual sessions and 3 optional group sessions) against the standard of care among PWH reporting hazardous alcohol consumption [12]. To be eligible for study participation, participants had to be ≥18 years old, living with HIV and receiving HIV care at 1 of 7 study-associated government ART clinics, and reporting hazardous alcohol consumption per the WHO Alcohol Use Disorders Identification Test Consumption (AUDIT-C; score ≥4 for men, score ≥3 for women) [13]. Persons were considered ineligible for the study if they were determined to be at risk of withdrawal (score ≥10 on the Clinical Institute Withdrawal Assessment) [14].

At the enrollment visit, participants completed a behavioral survey, as well as an interviewer-administered 30-day Timeline Follow-Back (TLFB) detailing their recent alcohol consumption [15, 16]. Standard drinks for each day were calculated based on the volume and alcohol content of each drink reported. Participants provided blood samples for a series of laboratory tests including a rapid HBV antigen test (Alere Determine HBsAg, Alere International Limited), a rapid HCV antibody test (SD BIOLINE HCV, Standard Diagnostics), and HIV viral loads (HIV-1 RNA test, COBAS AmpliPrep and COBAS TaqMan HIV-1 Test, Roche Molecular Systems). Referrals for HBV and HCV care were provided when relevant.

### Measures

During the enrollment study visit, a survey was administered on topics including demographics, HIV care information, drug use, alcohol use, and prior hepatitis diagnoses. These measures are included in this analysis as they are hypothesized to be associated with HIV/hepatitis coinfection [1, 4, 17]. Participants were asked if they had ever injected drugs and if they used drugs in the past three months (with or without injecting). Participants reporting a history of injection drug use were also asked whether they had ever tried to reduce frequency of injecting, and if so, whether they had increased their alcohol use while reducing injection drug use. The full AUDIT questionnaire was administered, with scores 8–14 indicating harmful alcohol use and scores ≥15 suggesting moderate-severe alcohol use disorder. A heavy drinking day was indicated as >4 drinks per day for men and >3 drinks per day for women on the TLFB. Participants were asked whether they had been previously diagnosed with hepatitis A, B, or C and if they were currently taking ART. HIV viral suppression was defined as a viral load <20 copies/mL.

### Statistical analysis

Descriptive statistics were used to characterize the study population. Participants were grouped by infection status into three groups: no coinfection (HIV only), one coinfection (either HBV *or* HCV), or two coinfections (both HBV *and* HCV). Initially, separate groupings were used to delineate participants with one coinfection (i.e., HIV and HBV *or* HIV and HCV); however, because few participants had HIV and HBV (without HCV), these groupings were combined for analysis. To explore both the magnitude and statistical significance of group differences, we conducted Fisher exact tests (given small cell counts of categorical variables) and Kruskal-Wallis rank sum tests (to account for nonnormal continuous variables). Characteristics that differed across coinfection groups (p<0.05) are presented by coinfection group and characteristics that did not differ across coinfection groups (p-value ≥0.05) are presented as a total. All analyses were conducted in R v4.1.1 (Vienna, Austria).

### Ethical approvals

All participants provided written informed consent for study participation and all study procedures were approved by the institutional review boards at the University of North Carolina-Chapel Hill, Johns Hopkins University, and Thai Nguyen Center for Preventative Medicine. The study is registered at clinicaltrials.gov, ID: NCT02720237.

## Results

A total of 440 participants living with HIV enrolled into the trial. Of those, 43 (10%) participants had no hepatitis coinfections, 355 (81%) had an HCV coinfection, 5 (1%) had an HBV coinfection, and 37 (8%) had HBV and HCV coinfections (Table 1).

**Table 1:** Baseline characteristics of participants by HIV/hepatitis coinfection status.

	Overall n (%)	Coinfection groups				
		No HBV or HCV n (%)	Either HBV or HCV n (%)	Both HBV and HCV n (%)	Group comparison p-value	
Characteristic						
**Total**	**440**	**43**	**360**	**37**	**--**	
Age in years, median (IQR)	40 (36–44)	39 (35–43)	40 (37–44)	39 (35–44)	0.19	
Sex					<0.01	
Male	426 (97)	32 (74)	357 (99)	37 (100)		
Female	14 (3)	11 (26)	3 (1)	0 (0)		
Currently taking ART					1.00	
Yes	438 (100)	43 (100)	358 (99)	37 (100)		
No	2 (0)	0 (0)	2 (1)	0 (0)		
Virally suppressed					0.47	
Yes	370 (84)	34 (79)	306 (85)	30 (81)		
No	70 (16)	9 (21)	54 (15)	7 (19)		
Prior diagnosis with hepatitis A					0.18	
Yes	15 (3)	1 (2)	11 (3)	3 (8)		
No	416 (95)	40 (93)	343 (95)	33 (89)		
Don’t know	9 (2)	2 (5)	6 (2)	1 (3)		
Prior diagnosis with hepatitis B					<0.01	
Yes	23 (5)	0 (0)	8 (2)	15 (41)		
No	407 (93)	41 (95)	345 (96)	21 (57)		
Don’t know	10 (2)	2 (5)	7 (2)	1 (3)		
Prior diagnosis with hepatitis C					0.01	
Yes	73 (17)	2 (5)	59 (16)	12 (32)		
No	357 (81)	39 (91)	294 (82)	24 (65)		
Don’t know	10 (2)	2 (5)	7 (2)	1 (3)		
Ever injected drugs in lifetime					<0.01	
Yes	356 (81)	3 (7)	317 (88)	36 (97)		
No	84 (19)	40 (93)	43 (12)	1 (3)		
Injection drug use in past 3 months^a^					<0.01	
Yes	118 (27)	0 (0)	102 (28)	16 (43)		
No	321 (73)	43 (100)	257 (71)	21 (57)		
Non-injection drug use in past 3 months					<0.01	
Yes	172 (39)	3 (7)	151 (42)	18 (49)		
No	268 (61)	40 (93)	209 (58)	19 (51)		
Ever tried to reduce injection drug use^a^					0.01	
Yes	269 (76)	1 (33)	235 (74)	33 (92)		
No	86 (24)	2 (67)	81 (26)	3 (8)		
N/A (never injected)	84	40	43	1		
Increased alcohol use while reducing injection drug use					0.20	
Yes	95 (35)	1 (100)	80 (34)	14 (42)		
No	174 (65)	0 (0)	155 (66)	19 (58)		
N/A (never injected or reduced)	170	42	124	4		
AUDIT score^a^, median (IQR)	12 (9–16)	10 (8–13)	12 (9–16)	11 (9–17)	0.01	

^a^Data were missing on injection drug use in the past 3 months for 1 participant; on attempts to reduce injection drug use for 1 participant; and on AUDIT score for 1 participant.

The median age of participants was 40 years old (interquartile range (IQR): 36, 44) and the vast majority of participants were male (n = 426 of 440, 97%; Table 1). There was a higher percentage of women in the no HBV or HCV group (n = 11 of 43, 26%) compared to the HBV or HCV(n = 3 of 360, 1%) and HBV and HCV groups (n = 0 of 37, 0%; p<0.01). Because recruitment occurred at ART clinics, nearly all participants reported taking ART (n = 438 of 440, 100%; p = 1.00), and most (n = 370 of 440, 84%; p = 0.47) were HIV virally suppressed.

Among the 42 participants who had a positive HBV surface antigen test at enrollment, 15 (36%) reported receiving a previous HBV diagnosis, all of whom were in the HBV and HCV group (15 of 37, 41%). Among the 392 participants who had a positive HCV antibody test at enrollment, 71 (18%) reported having been previously diagnosed with HCV (HCV only: n = 59 of 355, 17%; HBV and HCV: n = 12 of 37, 32%). Few participants in the entire study population (n = 15 of 440, 3%, p = 0.18) reported having been previously diagnosed with Hepatitis A.

We observed notable differences between coinfection groups in ever having injected drugs (p<0.01) and recent injection drug use (p<0.01; Table 1). Among those without hepatitis coinfection, few participants reported ever having injected drugs in their lifetime (n = 3 of 43, 7%) and none (n = 0 of 43, 0%) reported injection drug use in the last 3 months. In the HBV or HCV group, the majority (n = 317 of 360, 88%) of participants reported ever injecting drugs, with a minority (n = 102 of 360, 28%) reporting injecting drugs in the past 3 months. In the HBV and HCV group, nearly all participants reported ever injecting drugs (n = 36 of 37, 97%) with about half reporting injecting drugs in the past 3 months (n = 16 of 37, 43%). An increasing trend was observed across coinfection groups regarding non-injection drug use: 7% (n = 3 of 43), 42% (n = 151 of 360), and 49% (n = 18 of 37) of participants in the no HBV or HCV, HBV or HCV group, and HBV and HCV groups reported non-injection drug use in the past 3 months, respectively (p<0.01).

**Fig 1:**
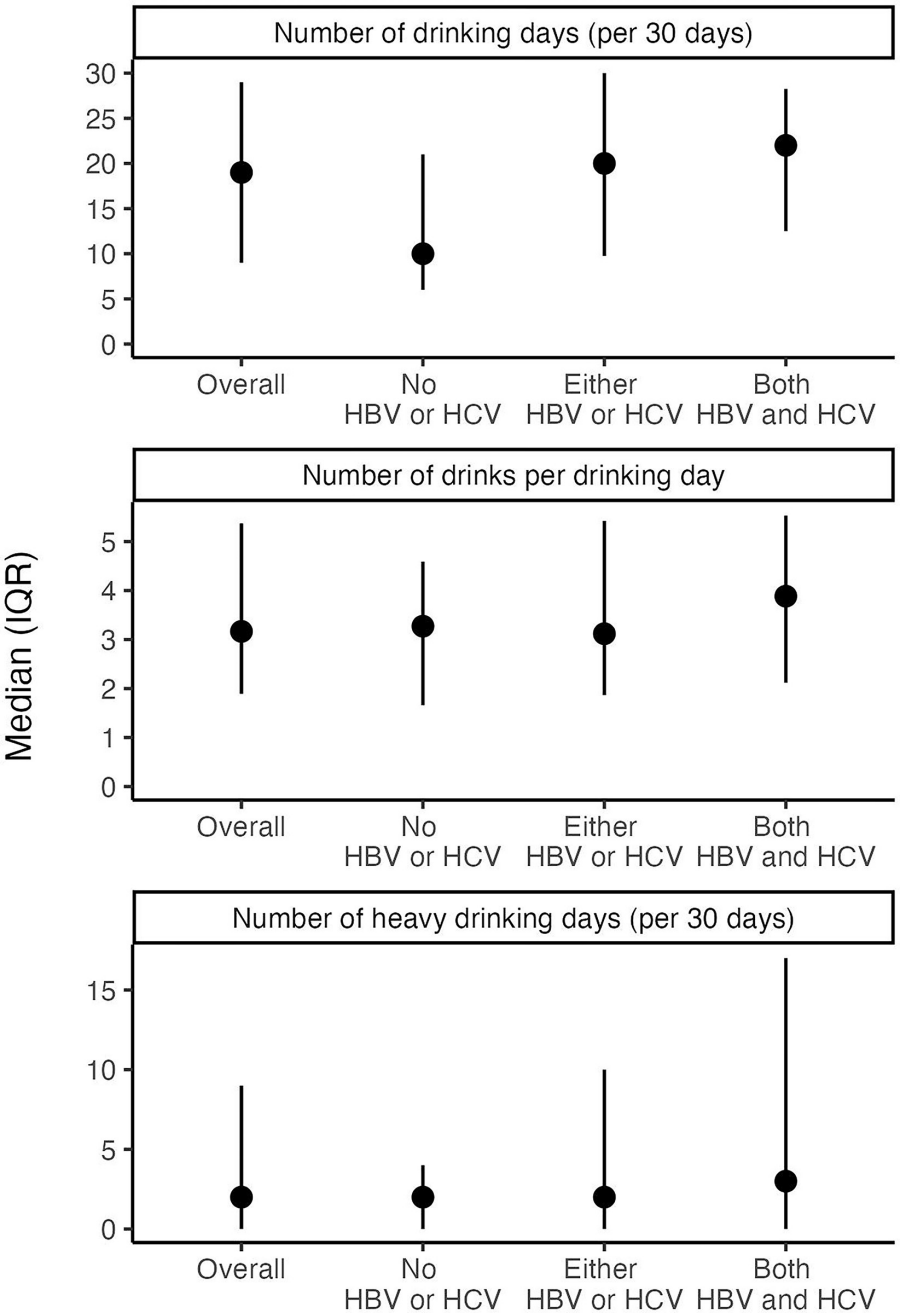
Participant alcohol use by HIV/hepatitis coinfection status.

Reported alcohol consumption across all three infection groups varied slightly with median AUDIT scores of 10 (IQR: 8, 13), 12 (IQR: 9, 16), and 11 (IQR: 9, 17) for the no HBV or HCV, HBV or HCV, and HBV and HCV, respectively (p = 0.01). Correspondingly, self-reported alcohol consumption from the past 30 days also differed slightly (Fig 1). Though the number of drinks on drinking days (median range: 3–4; p = 0.46) and the number of heavy drinking days (median range: 2–3; p = 0.66) were similar across groups, participants in the no HBV or HCV group reported a median of 10 (IQR: 6, 21) drinking days in the previous 30 days, while those in the HBV or HCV group reported a median of 20 (IQR: 10, 30) drinking days and those in the HBV and HCV group reported a median of 22 (IQR: 13, 28) drinking days (p<0.01).

Efforts to reduce injection drug use among those who had ever injected drugs varied across coinfection groups (p = 0.01), though there were no statistically significant differences when we looked at increasing alcohol while reducing injection drug use (p = 0.20). Among those who reported ever having injected drugs in the no HBV or HCV group, 1 of 3 (33%) reported trying to reduce injection drug use, and that person (100%) reported increasing alcohol use while reducing injection drug use. In the HBV or HCV group, 235 of 317 (74%) reported trying to reduce injection drug use, 80 (34%) of whom reported increasing alcohol use during those attempts. Among those in the HBV and HCV group who ever injected drugs, 33 of 36 (n = 92%) reported trying to reduce drug use, 14 (42%) of whom reported increasing alcohol use during their attempts.

## Discussion

Historically, HCV prevalence estimates in the general Vietnamese population range from 0.2–3.3% but have been much higher among those living with HIV (22.9–89.0%), as we saw in our population [18–21]. Both HIV and HCV can be transmitted through IDU, and while our sample limits our inferences to those living with HIV, our results support a confluence of HIV, HCV, and IDU as illustrated by the high prevalence of HCV among PWH and a higher reported prevalence of IDU among those with HIV and hepatitis coinfection [11]. Of note, HCV testing in our study was conducted with an antibody test, and we are unable to determine whether the positive results were from active or previously cleared infections. However, nearly 9 of 10 participants tested positive for HCV antibodies, indicating a high lifetime incidence of HCV in this population.

Prevalence of HBV coinfection in our study population was similar to estimates from the general population [9, 22, 23]. While HBV can be transmitted intravenously, in Vietnam, most incident HBV cases are thought to be transmitted perinatally [24]. Our sample size of persons with HBV and HIV (n = 5) did not enable us to examine differences in IDU compared to other coinfection statuses (HIV alone or HCV coinfection) and as such, we are unable to infer HBV-related behavioral associations from this population [24, 25]. However, understanding HIV and HBV coinfection is critical given the overlap in antiviral treatment: choosing antiviral regimens active against both HIV and HBV infections as well as any other concomitant condition is imperative for long term care [26].

Notably, among those who tested positive for HBV antigen and HCV antibodies, the majority had not received a diagnosis prior to the study. Only 36% and 18% of participants who tested positive had previously received a positive diagnosis for HBV and HCV, respectively. Screening for these infections is important particularly in this population given the high coinfection rates and benefits of early treatment [27]. Additionally, because participants were recruited from ART clinics, our results highlight the importance of this point of engagement in care for both coinfection testing and treatment referral as well as substance use assessment and outreach.

We observed more reported recent drinking days in the HBV or HCV group and HBV and HCV group as compared to the no HBV or HCV group. Furthermore, we found that among those who reported trying to reduce injection drug use, one in three reported increasing alcohol consumption during those attempts. Our data are cross-sectional and largely derived from self-report and thus we are unable to assess any causal or temporal relationships. Furthermore, our study population limits our inferences to PWH seeking ART care and reporting hazardous alcohol use. However, the confluence of alcohol use and hepatitis coinfection in this population highlights the entwined nature of these syndemic conditions and the potential for increased hepatic morbidity. As HIV coinfection has shown to hasten liver disease progression among those with HCV, and hazardous alcohol consumption increases the risk of cirrhosis and fibrosis, cooccurring HCV, HIV, and hazardous alcohol use have the potential for a synergistically negative effects [28, 29]. Therapies for HBV and HCV are becoming more widely available in Vietnam, and successful interventions for alcohol and drug use have been developed and adapted locally [12, 26, 30, 31]. Integrating screening and treatment for these syndemic conditions as coordinated care will benefit patient care and prognostic outcomes [27].

The syndemics of HIV, hepatitis, IDU, and alcohol use are deeply entangled and challenging to parse out. However, the differences we observed between coinfection groups highlights patterns of substance use within a vulnerable population. Future research should assess barriers to substance use and hepatitis screening and care referral programs in the HIV care setting in Vietnam.

## Supporting information

S1 Dataset Deidentified dataset for “Hepatitis and HIV coinfection among hazardous alcohol users in Vietnam”. (CSV)
